# Phylogenetic allometric scaling of near basal breathing frequency in terrestrial, semi‐aquatic and aquatic mammals

**DOI:** 10.1113/EP091868

**Published:** 2025-04-01

**Authors:** Andreas Fahlman, Elliot Stielstra, Ethan Wilstermann, Simon Rylaarsdam, Josefin Larsson, Guillermo J. Sanchez‐Contreras, Suguru Higa, Gonçalo N. Marques, Malgosia Kaczmarska, Jason Somarelli, Stacy L. DeRuiter

**Affiliations:** ^1^ Research Department Fundación Oceanogràfic Valencia Spain; ^2^ Global Diving Research San Lucar de Barrameda Spain; ^3^ IFM Linkoping University Linkoping Sweden; ^4^ Department of Mathematics and Statistics Calvin University Grand Rapids Michigan USA; ^5^ Kolmården Wildlife Park Kolmården Sweden; ^6^ Veterinary Department The Dolphin Company Cancún Mexico; ^7^ Marine Mammal Department Okinawa Churaumi Aquarium Okinawa Japan; ^8^ Veterinary Department Zoomarine Algarve Guia Portugal; ^9^ Zoo d'Amnéville Amneville France; ^10^ Department of Medicine Duke University Medical School Raleigh North Carolina USA

**Keywords:** metabolic rate, respiration, ventilation

## Abstract

We measured the BASAL breathing frequency following an overnight fast in adult, non‐pregnant/non‐lactating, inactive mammals ranging in body mass from 15 to 5520 kg. The data included results from 338 individual animals from 34 species that were divided into terrestrial, semi‐aquatic (*Otariidae* and *Phocidae*) and aquatic mammals. Following attempts to limit the collection of breathing frequency using a basal definition and to correct the analysis phylogenetically, our results suggest that there are differences in the allometric mass‐exponent between terrestrial and aquatic/semi‐aquatic mammals. An allometric regression model, whereby both body mass and breathing frequency were transformed using log_10_, suggested that the allometric mass exponent for terrestrial mammals (−0.303) was different from both aquatic mammals (−0.124) and semi‐aquatic mammals (−0.091). For semi‐aquatic mammals, the breathing frequency was lower in water, but we detected no association between the breathing frequency and the temperature of the medium (water or air). We propose that allometric studies of cardiorespiratory function should, if possible, adhere to the basal definition during data collection, similar to that used for metabolic rate. Such data will provide valuable information for comparative medicine of large species that are difficult to study, for which controlled baseline data might be difficult to obtain.

## Abstract (highlights)

- **What is the central question of this study?**
  In this study, we examine the relationship between breathing frequency and body mass in mammals from different habitats (aquatic, semi‐aquatic and terrestrial), accounting for phylogeny.
- **What is the main finding and its importance?**
  Similar to past studies, the present results suggest that the respiratory frequency scales differently in aquatic/semi‐aquatic mammals compared with terrestrial mammals. However, the mass‐exponent (slope) for aquatic/semi‐aquatic mammals is less steep in comparison to past studies.

## 1 INTRODUCTION

The term allometric scaling was coined by Julian Huxley and Georges Tessier in 1936 (Huxley & Teissier, 1936), but the study of the relationship between body size and morphology or physiology dates to the mid‐19th century, when Max Rubner (Rubner, 1865) provided the first definition of basal metabolic rate (BMR) and Otto Snell (Snell, 1892) outlined the relationship between brain weight and body size. The relationship between body size and BMR, also often referred to as Kleiber's law, was published by Max Kleiber (Kleiber, 1932), who showed that energy requirements are correlated non‐linearly with body mass (*M*_b_) to the power of 0.75, commonly called the allometric scaling factor. There have been numerous publications that debate the value of the allometric scaling factor, but it remains frequently discussed, and it has been shown to vary with conditions such as diet and habitat/environment (He et al., 2023; McNab, 2008, 2009; White, 2010; White et al., 2007).

Several variables are known to alter metabolic rate, including digestion (called the heat increment of feeding or specific dynamic action) and thermoregulation in an environment with a temperature outside the thermoneutral zone (Metze, 2016; Secor, 2009). The definition of ‘basal’ metabolic rate restricts measurements to adult, non‐pregnant and not lactating, postprandial animals that are inactive/resting, but not sleeping, and are in their thermoneutral range (Kleiber, 1932). Comparing metabolic rates between individuals or species that are not measured using the basal definition, therefore, increases variation and might lead to erroneous conclusions.

Aerobic metabolism depends on two convective steps to supply the cell with O_2_: ventilation [breathing frequency (*f*_R_) and tidal volume] and cardiac output (heart rate and stroke volume). Therefore, not surprisingly, similar scaling relationships have been shown for both ventilation and perfusion (He et al., 2023; Seymour & Blaylock, 2000; Stahl, 1967). However, unlike BMR, cardiorespiratory variables, such as tidal volume, breathing frequency, heart rate or stroke volume, are seldom standardized, and some studies include anaesthetized or restrained animals or average daily values where the animals have varying activity (He et al., 2023; Mortola & Limoges, 2006; Seymour & Blaylock, 2000; Stahl, 1967). In smaller cetaceans, where digestive processes can increase metabolic rate as much as 40% above BMR following a standard meal (Fahlman et al., 2024; Yeates & Houser, 2008), it was shown that the average heart rate in fed bottlenose dolphins increased by between 9% and 14% as compared with after an overnight fast, after accounting for breathing frequency (Blawas et al., 2021).

In recent studies, it has been shown that the allometric scaling factor for breathing frequency is different in terrestrial and aquatic species (He et al., 2023; Mortola & Limoges, 2006). Given that these published studies used data that were not measured in basal conditions, it is difficult to evaluate the potential variation around each data point and the conclusions from those studies. To gain a better understanding of how breathing frequency varies with metabolic demands (Fahlman et al., 2016; Roos et al., 2016; Videsen et al., 2023), the cardiorespiratory coupling (Fahlman, 2024; Mortola, 2015), and also as a tool to diagnose respiratory health (Butterworth et al., 2004), it is of interest to define how breathing frequency scales between species and varies for species that inhabit different habitats, such as the terrestrial versus aquatic environment (Agostoni et al., 1959). Therefore, in this study the aim was to collect data on respiratory frequency in awake, unrestrained, adult, fasted mammals. Specifically, we wanted to assess the allometric relationship among terrestrial, semi‐aquatic (*Otariidae* and *Phocidae*) and aquatic mammals in near‐basal conditions and to correct for phylogeny.

## 2 MATERIALS AND METHODS

### 2.1 Ethical approval

The data collection in the present study was done by focal observations that did not impact the animals or their behaviour (e.g., there was no habitat alteration, offering food or nesting material choices, calling or baiting), and each participating facility approved the collection of respiratory data from the species in their facility. In addition, focal observations to collect breathing frequency were part of the daily husbandry routine in all participating institutions, and the only alteration was the time of data collection to ensure that measurements could be performed in fasted animals without altering their normal feeding time. As such, observations were performed before the normal time when the animals were fed in the morning, and all animals always had access to water. Approval of the observations was also provided by an official animal welfare committee at the Oceanografic (Approval #OCE‐1‐23).

### 2.2 Data collection

A request was sent to different facilities that house mammals in professional care to collect breathing frequency through focal observations in awake, non‐restrained, adult, postprandial (after an overnight fast) and non‐pregnant mammals at rest. Given that opportunistic focal observations could not guarantee that all animals were at rest, the observer was asked to score the activity in three levels: (1) rest/inactive; (2) minimal activity; and (3) active. All observers had several years of experience in handling and working with the species for which they measured the breathing frequency and had previously measured breathing frequency for daily husbandry records.

Data were received from a total of 23 zoological institutions from a total of 1240 measurements in 338 individual animals from 34 species (and one subspecies of bottlenose dolphin) ranging in body mass from 15 to 5270 kg (Table 1). Data included the common name (and taxonomic order, family, genus and species), animal identity, sex, body mass, year of birth (for wild‐caught individuals, the age was estimated), activity level during focal observation, number of breaths and duration of measurement, whether measured in water or on land (for semi‐aquatic species), temperature of the environment where measured (water and/or air), institution and date of measurement. Phylogenetic relationships between taxa were inferred using TimeTree (Kumar et al., 2022) and exported in Newick format for visualization in FigTree. Heat map annotations were generated in Morpheus and coloured by the ratio of the common logarithm (log_10_) of breathing frequency and body mass [log_10_(*f*_R_) log_10_(*M*_b_)^−1^] for each species.

**TABLE 1:** Summary of all orders, genera and species of terrestrial, semi‐aquatic and aquatic mammals, with their range of body mass and breathing frequency.

Order	Family	Genus	Species	Body mass (kg)	Breathing frequency (breaths min^−1^)
Artiodactyla	6	15	16^(6,0,9)^	35.5–3690	0.2–45.2
Carnivora	4	10	13^(4,9,0)^	15–939	0.6–42.6
Perissodactyla	2	2	2^(2,0,0)^	184–2200	4.4–22.5
Proboscidea	1	1	1^(1,0,0)^	3340–5570	1.8–9.7
Rodentia	1	1	1^(1,0,0)^	43–55	22.8–44.3
Sirenia	1	1	2^(0,0,2)^	200–2000	0.2–1.6
All	15	30	34^(14,9,11)^	15–5570	0.2–45.2

Superscripted numbers in parentheses represent the number of terrestrial, semi‐aquatic and aquatic species, respectively, for each order.

### 2.3 Statistical analysis

We used a linear mixed model in R (package glmmTMB, Brooks et al. 2017), using breathing frequency (*f*_R_) as a dependent variable and with habitat (aquatic, semi‐aquatic or terrestrial), environmental temperature (air or water; for some semi‐aquatic mammals, measurements were made both in air and in water, and the temperature where the individual was measured was used), activity level (rest, some activity or active) and body mass (*M*_b_) as independent variables. Both *f*_R_ and *M*_b_ were transformed using log_10_. We used a hierarchical model, including a random effect of individual identity and nested random effects of order, family, genus and species. We used a type II ANOVA to test for associations between breath frequency and independent variables (function Anova from the car package in R; Fox & Weisberg, 2019), with a *post hoc* test to assess variation in slopes between habitats (function emtrends in the package emmeans in R; Lenth et al., 2022).

## 3 RESULTS

The results from the statistical analysis are presented in Table 2, and the *post hoc* testing for differences in slopes between habitat and body mass is detailed in Table 3.

**TABLE 2:** Parameter estimate (±SE), *z*‐value (and corresponding *P*‐value), χ^2^ for type II Wald statistics (and corresponding *P*‐value) from the linear mixed model with log_10_‐transformed breathing frequency (in breathsper minute^;^ log_10_ *f*_R_) as the dependent variable and with log_10_‐transformed body mass (in kilograms; log_10_ *M*_b_) and temperature (in degrees Celsius) as independent continuous variables, and with habitat (terrestrial, semi‐aquatic and aquatic), activity level (1, rest; 2, limited/minimal activity; 3, active), location (whether the individual was measured on land or in water) and the interaction between log_10_ *M*_b_ and habitat to test for differences in slopes.

Parameter		Parameter estimate ± SE	*Z*‐value (*P*‐value)	χ^2^ (*P*‐value)
Intercept		0.489 ± 0.150	3.254 (0.0011)	
log_10_ *M*_b_		−0.114 ± 0.044	−2.610 (0.0091)	29.2 (< 0.0001)
Habitat	Semi‐aquatic	0.185 ± 0.174	1.062 (0.2881)	201.1 (< 0.0001)
	Terrestrial	1.299 ± 0.189	6.867 (< 0.0001)	
Activity level	2	0.082 ± 0.010	8.246 (< 0.0001)	120.8 (< 0.0001)
	3	0.172 ±0.017	9.950 (< 0.0001)	
Location_water_		−0.067 ± 0.024	−2.811 (0.0049)	7.9 (0.0049)
Temperature		−0.0002 ± 0.0011	−0.199 (0.843)	0.039 (0.843)
log_10_ *M*_b_ × habitat	Semi‐aquatic	0.013 ± 0.067	0.190 (0.849)	9.1 (0.010)
	Terrestrial	−0.201 ± 0.074	−2.711 (0.007)	

**TABLE 3:** Allometric mass‐exponent (±SE) for each habitat and *post hoc* testing (*t*‐ratio and *P*‐value) for differences in slopes between log_10_‐transformed body mass and habitat (terrestrial, semi‐aquatic and aquatic).

Habitat	Parameter estimate ± SE	*t*‐ratio (*P*‐value) comparing habitats	
		Terrestrial	Semi‐aquatic
Terrestrial	−0.315 ± 0.060		
Semi‐aquatic	−0.101 ± 0.051	2.720 (0.018)	
Aquatic	−0.114 ± 0.044	2.711 (0.019)	−0.013 (0.980)

The results indicate that there is an allometric relationship between body mass and breathing frequency (Table 2). The full model passed assessment, including checks for linearity and for normality, independence and constant variance of residuals. Supplementary material, including R code and access to all data, are available at: https://stacyderuiter.github.io/breath‐allometry/supplemental‐materials.html

The results indicate that there were differences in the slope of the breathing rate–mass relationship between aquatic/semi‐aquatic and terrestrial mammals, but not between aquatic and semi‐aquatic mammals (Tables 2 and 3). The estimated allometric slopes for aquatic and semi‐aquatic mammals were −0.114 (95% confidence interval: −0.199 to −0.028) and −0.101 (95% confidence interval: −0.201 to −0.002), and that for terrestrial mammals was −0.315 (95% confidence interval: −0.433 to −0.197) (computed using the function *emtrends* from the R package *emmeans*). Breathing frequency increased with increasing activity level but was not affected by the temperature of the environment (water or air) where the individual was measured.

**FIGURE 1:**
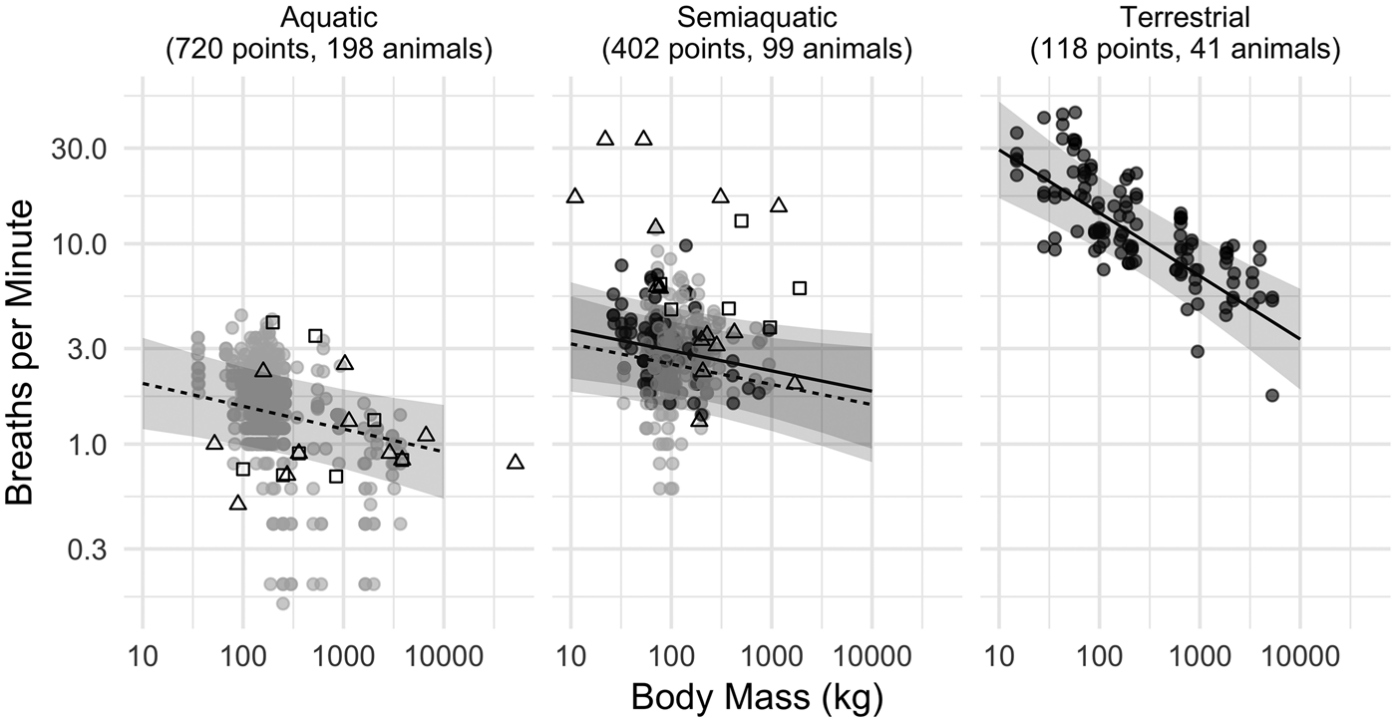
Common logarithm (log_10_)‐transformed breathing frequency (in breaths per minute) against body mass (in kilograms) for aquatic (left), semi‐aquatic (middle) and terrestrial (right) mammals for 1240 measurements from 338 individual animals representing six orders and 34 species of mammals (number of measurements [point] and individual animals are inside parentheses). Grey dots show measurements taken in water and black on land. Dashed lines indicate model predictions for mammals in water (activity level 1), and continuous lines in air (activity level 1), with grey bands showing the 95% confidence intervals. On the plot are also data from He et al. (2023) in open squares, and from Mortola & Limoges (2006) in open triangles. An interactive version of this figure is available in the online supplementary materials, which allows users to identify data points by order, species and individual.

Figure 1 shows breathing frequency plotted against body mass, separated by habitat (with lines showing expected breathing rates at rest, i.e., an activity level of 1). In this figure are also plotted values from two previously published studies that investigated the relationship between breathing frequency and body mass in terrestrial and aquatic mammals (He et al., 2023; Mortola & Limoges, 2006).

**FIGURE 2:**
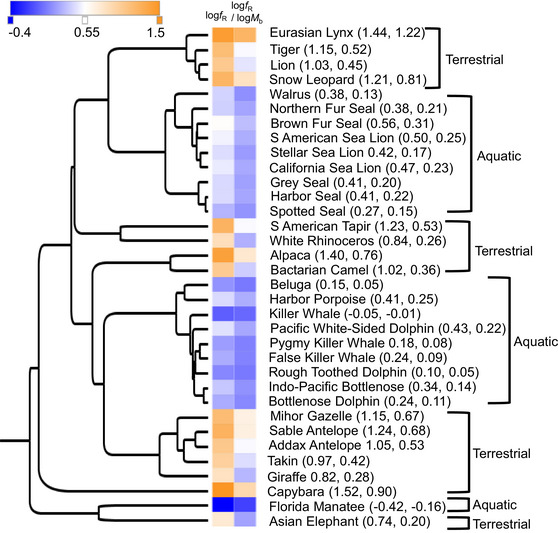
Basal respiration across taxa. Basal breathing frequency (*f*_R_) and body mass (*M*_b_)‐corrected respiration [log_10_(*f*_R_) × log_10_(*M*_b_)^−1^] are indicated as a heat map along a phylogeny inferred by TimeTree. Average log_10_(*f*_R_) and log_10_(*f*_R_) × log_10_(*M*_b_)^−1^, respectively, are indicated in parentheses next to the common name for each species.

Analysis of body mass‐corrected breathing frequencies in relationship to phylogenetic relationships between species revealed distinct distributions of breathing frequencies across diverse phylogenetic clades (Figure 2). Differences in breathing frequencies were stratified based on aquatic or terrestrial habitats, and this was true for terrestrial and aquatic organisms within the same clade. This is most evident within the artiodactyls, in which bovines, camelids and giraffes exhibit higher log_10_‐transformed mass‐corrected breathing frequency (log_10_ *f*_R_/log_10_ *M*_b_) (mean ± SD = 0.51 ± 0.18) than the clade composed of the delphinids, monodontids and Phocoenidae (porpoises) (mean = 0.11 ± 0.09) (Figure 2).

## 4 DISCUSSION

In the present study, we collected breathing frequency from 338 unrestrained individual terrestrial, semi‐aquatic and aquatic mammals following an overnight fast. Although focal collection of breathing frequency included measurements of both resting and active individuals at different environmental temperatures, the analysis enabled us to estimate breathing frequency for inactive mammals (activity level 1). These data provide near‐basal estimates of breathing frequency in mammals, and the analysis has been corrected for phylogeny. Our results suggest that the relationship between breathing frequency and body mass is lower in aquatic and semi‐aquatic mammals than proposed in past studies.

Allometric scaling is often used to compare energetic needs across species of different body sizes (He et al., 2023; McNab, 2008, 2009; White, 2010; White et al., 2007). Such studies commonly use data from the published literature that have measured BMR, which helps to reduce variation attributable to confounding factors, which is helpful in comparative studies. The allometric mass component, the slope of the relationship between body mass and metabolic rate, ranges between 0.66 and 0.75 (White, 2010). Given that increased aerobic metabolism results in elevated convective demand for O_2_, similar relationships have been shown to exist for ventilation and perfusion, and both minute ventilation (breathing frequency × tidal volume) and cardiac output (heart rate × stroke volume) scale with a similar mass‐exponent to that for BMR (He et al., 2023; Seymour & Blaylock, 2000; Stahl, 1967). Despite differences in the breathing strategy between aquatic and terrestrial mammals, whereby terrestrial mammals generally have a higher breathing frequency and lower tidal volume in comparison to aquatic mammals (Fahlman et al., 2017; He et al., 2023; Mortola & Limoges, 2006; Piscitelli‐Doshkov et al., 2024), the minute volume has been shown to scale with metabolic rate in both groups, that is, with an allometric mass‐exponent of ∼0.66–0.75. However, given that few comparative studies on cardiorespiratory physiology have attempted to control for variables that might alter metabolic rate, the potential confounding effects of digestion, age and activity are not known. For example, it was shown that dolphins that had fasted overnight had a lower heart rate than those that had been fed recently (Blawas et al., 2021). Thus, studies that have not controlled for potential conflicting effects could suffer from increased variability, potentially leading to erroneous conclusions.

The main objective of this study was to determine the allometric relationship between breathing frequency and body mass for mammals that reside in different habitats by restricting the data to basal measurements, that is, postprandial, adult individuals, at rest and in their thermoneutral environment. Although BMR is a well‐defined unit that allows comparison between species of different sizes and that reside in different habitats, a similar definition is not commonly made for physiological variables that are known to vary with metabolic rate, such as breathing frequency. In controlling for these potential confounding variables, we hypothesized that the data presented in the present study would be less variable in comparison to past studies (see Figure 1; He et al., 2023; Mortola & Limoges, 2006). Although we could not make a direct comparison, our results show, for example, that for semi‐aquatic mammals, the breathing frequency differs for animals on land or in water. Given that past studies have not analysed these separately, this would have increased the variation. In addition, the breathing frequency in the present study was lower overall in comparison to those presented in past studies (Figure 1). Although the results for terrestrial mammals showed a correlation between body mass and breathing frequency, the data from both semi‐aquatic and aquatic mammals were as variable as those presented in past studies, even when accounting for activity (Figure 1). In addition, the limited body mass range of mammals included in this study might have affected the results, especially the lack of otter species, which seems to skew the relationship positively. For aquatic mammals, in contrast, our model predictions appear to extend accurately to match fin whale data from a previous study (Figure 1). Thus, our results indicate that body mass by itself might exhibit only a weak relationship to breathing frequency in semi‐aquatic and aquatic mammals, and other variables, such as habitat, life history, season etc., might be important confounding variables.

Focal observations, such as those presented here, allow the breathing frequency to be measured with minimal interference to the individual animal. However, during such observations it is not always possible to provide measurements of animals at a specific activity level. One option would be to reduce the data set to include only animals at rest. We chose to include all observations and recorded the observed activity level (a separate analysis of the restricted data set provided equivalent results) but then displayed model predictions only for animals at rest (Figure 1). Although we attempted to measure all animals while postprandial, true BMR might not be possible for species such as ruminants, which can require up to7 days to be postprandial (Baxter, 1967; McNab, 1997; National Research Council Subcommittee on Environmental, 1981), or for certain species of carnivores that consume a large meal, then fast for several days. For all species, we therefore made the measurements before they were fed in the morning, following an overnight fast. Although this might not be a sufficiently long fasting period for some species, this was the longest period that was justified to prioritize animal welfare. In addition, the breathing frequency of the ruminants in the present study did not differ noticeably from other groups (Figure 1). Finally, although we measured each individual and species in an environment where they had been housed over an extended period of time, we could not be sure that all species were in their thermoneutral range. One reason for this is that the true thermoneutral range has not been measured in many species. The data therefore included the measurement temperature and the range of temperatures that each species is known to experience in their normal habitat (see data in the Data availability statement). However, unlike past studies, we found the allometric scaling factor for aquatic and semi‐aquatic species to be considerably lower than previously reported.

In two past studies, it was shown that the allometric scaling factor for breathing frequency for aquatic species had a steeper negative slope (−0.34 to −0.42) in comparison to terrestrial mammals (−0.24 to −0.25) (He et al., 2023; Mortola & Limoges, 2006). In the present study, the allometric mass exponent for breathing frequency was not different for aquatic and semi‐aquatic mammals, but the mass exponent for aquatic and semi‐aquatic mammals was lower than for terrestrial mammals (Tables 2 and 3). In addition, the breathing frequency was lower in semi‐aquatic mammals when measured in water compared with on land (Table 2). There are several potential reasons for these differences. In past studies, data collection was not controlled for the fasting state and came from a wide range of sources, such as animals in human care, laboratory studies and results from wild animals, where the fasting state, whether an animal is awake or anaesthetized, the body mass, age/maturity or pregnancy status are seldom reported (He et al., 2023; Mortola & Limoges, 2006). Another potential reason could be how the species were separated into different habitats. For example, in the present study, we did not consider the capybara (*Hydrochoerus hydrochaeris*) to be an aquatic species, and the data set in the present study did not include any otter species (*Enhydra* spp.) or the polar bear (*Ursus maritimus*; Figure 1). Given that these species had higher breathing frequencies than expected from the results in the present study (see Figure 1, semi‐aquatic), their inclusion might have increased the slope of the relationship in the past studies. When overlaying the results from the past studies on the regression line obtained in the present study, it can be observed that most data points used in the earlier studies fall above the regression lines, especially for semi‐aquatic mammals (Figure 1).

We plotted the basal breathing frequency along phylogeny, using a colour gradient (heat map) to indicate variation in body mass‐adjusted breathing frequency (Figure 2). The figure shows that habitat (aquatic/semi‐aquatic vs. terrestrial) is a stronger predictor than phylogeny for breathing frequency. Thus, the aquatic breathing strategy (Fahlman et al., 2017), with an exhalation followed by inhalation and with a long inter‐breath interval, appears to be conserved among species that are either fully or semi‐aquatic. Whether this represents convergent evolution is not clear, but breathing strategy might be a highly plastic trait, and the data from the semi‐aquatic species in the present study support this, because their breathing frequency decreases in water. Furthermore, both humans entering water and human neonates (te Pas et al., 2009) reverse their breathing pattern to resemble aquatic breathing.

The allometric scaling of breathing frequency examined in this study highlights how different species meet the convective demands required to supply sufficient oxygen for aerobic metabolism. Consistent with previous research, the findings confirm that aquatic and semi‐aquatic species exhibit lower mass‐corrected breathing frequencies in comparison to terrestrial mammals (He et al., 2023; Mortola & Limoges, 2006). To maintain adequate convective requirements, these species compensate by increasing tidal volume to ensure constant alveolar ventilation. Studies measuring tidal volume in aquatic and semi‐aquatic mammals have shown that these species breathe with a mass‐corrected tidal volume greater than that of terrestrial mammals, typically 30%–40% of their estimated vital capacity (Fahlman et al., 2020, 2017).

This breathing strategy might enhance the alveolar–arterial gradient for oxygen and carbon dioxide, improving the efficiency of gas exchange and facilitating recovery during surface intervals between dives (Fahlman et al., 2008). However, evidence is mixed regarding whether this strategy significantly affects the alveolar–arterial oxygen difference (Fahlman et al., 2015, 2024; Mortola & Limoges, 2006). Some studies suggest that lung volume plays a more crucial role in buoyancy control rather than gas exchange (Mortola & Limoges, 2006). Further research on breathing frequency, tidal volume and alveolar–arterial gas gradients is necessary to understand fully how respiratory adaptations support metabolism in aquatic and semi‐aquatic mammals.

The results presented in the present study highlight the importance of carefully selecting the data depending on the research question. For example, the allometric scaling of BMR is used in comparative veterinary medicine to estimate drug dosage in species for which there is little or no information. However, during general anaesthesia, the mode of ventilation and the anaesthetic dose can also depend on the mode of breathing of the individual species (Le‐Bert et al., 2024). For example, adapting the manual ventilation mode for the bottlenose dolphin helped to improve ventilation and gas exchange during anaesthesia (Le‐Bert et al., 2024), because this species has a lower breathing frequency and higher tidal volume in comparison to terrestrial mammals, breaths that begin with exhalation followed by inhalation, and long inter‐breathing intervals (Fahlman et al., 2017; Piscitelli‐Doshkov et al., 2024).

The results presented here highlight how comparative respiratory physiology can be useful for comparative medicine and wildlife conservation. Breathing frequency is relatively easy to measure and is a potentially useful index for respiratory health and overall stress (Divers, 2008). However, this requires measurements that define baseline values, which can be challenging logistically when dealing with larger, cryptic and difficult‐to‐study species (He et al., 2023). In the present study, collaboration with several institutions that house terrestrial, semi‐aquatic and aquatic mammals permitted non‐invasive data collection, which provided baseline respiratory information that could be used to evaluate respiratory health in wild species. For example, respiratory disease is one of the most common causes of morbidity and mortality in cetaceans, both in wild animals and in those housed in managed care (Sharp et al., 2014; Sweeney & Ridgway, 1975; Venn‐Watson et al., 2012; Fahlman et al., 2025). Respiratory disease is often masked, and symptoms often occur when the disease has progressed to an advanced stage. Thus, breathing frequency might be a simple and useful method to evaluate respiratory health and stress. In poorly studied species, allometric extrapolation from related wild species might complement wildlife health assessments, including habitat quality monitoring, the impacts of climate change and human activities, and for early management of potential disease outbreaks.

From a clinical point of view, empirical extrapolation of medical protocols or drug delivery is already common practice but might be problematic when extrapolating between species (Freitas & Carregaro, 2013; Mahmood, 2007). Clinicians often use allometric scaling of BMR to estimate a drug dose or for other clinical assessments, whereas species‐specific physiological details, such as respiratory rate, are often ignored (Freitas & Carregaro, 2013; Mahmood, 2007). The allometric relationship presented here, using data collected on animals with a basal definition, provides a complementary method to evaluate baseline respiratory physiology that reduces the risk of medical extrapolation, improving safety and efficacy. Moreover, understanding how breathing frequency scales with size between species permits an initial evaluation of respiratory and systemic health. This can be particularly valuable in a field setting, where rapid triage and prioritization are required before a more detailed comprehensive diagnosis of respiratory or other underlying disorders is possible.

## 5 CONCLUSION

In the present study, we collected breathing frequency the morning after an overnight fast in adult, inactive mammal species over a body mass range of 2.5 orders of magnitude. We divided the mammals into terrestrial, semi‐aquatic and aquatic categories based on their lifestyle. The results suggest that there are differences in the allometric relationship between breathing frequency and body mass, with a different slope (allometric constant) between aquatic/semi‐aquatic mammals and terrestrial mammals, but not between aquatic and semi‐aquatic mammals. The results in aquatic/semi‐aquatic mammals differ substantially from previous studies, which might reflect differences in studies on fed and fasted mammals. These data might provide useful baseline values for estimating respiratory frequency in difficult studies and in large species, in which controlled studies are difficult, and could help with conservation management of threatened species.

## AUTHOR CONTRIBUTIONS

All authors contributed to the conception or design of the work, the acquisition, analysis or interpretation of data for the work, and the drafting, commenting or editing of the article. All authors approved the final version of the manuscript and agreed to be accountable for all aspects of the work in ensuring that questions related to the accuracy or integrity of any part of the work are appropriately investigated and resolved. All persons designated as authors qualify for authorship, and all those who qualify for authorship are listed.

## CONFLICT OF INTEREST

None declared.

## FUNDING INFORMATION

None.

## Data Availability

Supplementary materials, including R code and access to all data, are available at: https://stacyderuiter.github.io/breath‐allometry/supplemental‐materials.html.
